# New Applications of Photodynamic Therapy in Biomedicine and Biotechnology

**DOI:** 10.1155/2013/161362

**Published:** 2013-05-28

**Authors:** Kristjan Plaetzer, Mark Berneburg, Tobias Kiesslich, Tim Maisch

**Affiliations:** ^1^Laboratory of Photodynamic Inactivation of Microorganisms, Department of Materials Science and Physics, University of Salzburg, Hellbrunnerstraße 34, 5020 Salzburg, Austria; ^2^Department of Dermatology, Eberhard Karls University, Liebermeisterstraße 25, 72076 Tuebingen, Germany; ^3^Department of Internal Medicine I, Paracelsus Medical University and Salzburger Landeskliniken (SALK), Muellner Hauptstrasse 48, 5020 Salzburg, Austria; ^4^Institute of Physiology and Pathophysiology, Paracelsus Medical University, Strubergasse 21, 5020 Salzburg, Austria; ^5^Department of Dermatology, University Hospital Regensburg, Franz-Josef-Strauss-Allee 11, 93053 Regensburg, Germany

Photodynamic procedures are based on the light-induced, photosensitizer-mediated overproduction of reactive oxygen species for removal of harmful or unwanted cells/pathogens. With approvals for various applications by health agencies in most industrial countries, Photodynamic Therapy (PDT) represents the method of choice for treatment of age-related macular degeneration and is appreciated as minimally invasive therapeutic procedure to treat skin, esophageal, head and neck, lung, and bladder cancers with high cure rates, low side effects, and excellent cosmetic outcome. Being motivated by the success of PDT in management of various human diseases, new and very promising applications of this procedure are being identified and explored by the PDT research community. So, for example, Photodynamic Inactivation of microorganisms (PDI) has the potential to avert the severe threat of increasing antimicrobial resistance. This special issue presents a collection of papers dealing with novel photosensitizers with improved photochemical and photophysical properties, alternatives to (single) photon excitation of the photoactive drug, new clinical targets of photodynamic procedures, and innovative reviews on crucial aspects, which substantiate the success of Photodynamic Therapy and Photodynamic Inactivation.

In a research paper entitled “*Hydrogen bond acceptors and additional cationic charges in methylene blue derivatives: photophysics and antimicrobial efficiency*,” A. Felgenträger et al. report on the synthesis and photodynamic efficiency of six novel methylene blue derivatives with highly polar and/or hydrophilic groups. Based on a systematic analysis of singlet oxygen formation in combination with the absorbed light energy and on investigations of the phototoxicity towards *Staphylococcus aureus* and *Escherichia coli*, a structure-activity relationship has been drawn from a chemical point of view. The data presented in this study demonstrate that hydrogen acceptor bond moieties and additional tertiary charges in the substituent have a positive influence on the overall antimicrobial efficacy of methylene blue derivatives, resulting in a convincing inactivation of viable Gram (+) or Gram (−) bacteria up to 7 log units.

Conjugates of nanoparticles and photosensitizers represent a very promising new generation of photoactive substances. The correlation of the size of purpurin-18-*N*-methyl-*D*-glucamine gold nanoparticles and the respective photodynamic activity is described by B. Lkhagvadulam et al.'s “*Size-dependent photodynamic activity of gold nanoparticles conjugate of water soluble purpurin-18-N-methyl-D-glucamine*”. The authors demonstrate that the PDT efficiency of photosensitizer-nanoparticle conjugates against A549 lung cancer cells is higher when compared to the unbound photosensitizer because of the increased internalization of the PS into the target cells using the size effect. The highest photodynamic activity is reported to be associated with rather bigger conjugates with a diameter of about 60 nm.

Y-.S. Chou et al.'s “*Photo-induced antitumor effect of 3,6-Bis(1-methyl-4-vinylpyridinium) carbazole diiodide*” evaluated the photo-induced antitumor effect of 3,6-bis(1-methyl-4-vinylpyridinium) carbazole diiodide (BMVC) in a lung tumor cell line (TC-1) and the corresponding *in vivo* murine model (TC-1 cell tumors in C57BL/6 mice). BMVC is able to induce a phototoxic effect in TC-1 cells at light fluences greater than 40 J/cm² and significantly reduced the tumor growth rate *in vivo* following fine-needle interstitial light illumination of the photosensitizer. Immunohistochemistry studies showed that the microvascular density was lower not only in the PDT group but also in animals, which received light only (without photosensitizer). Therefore, the antivascular effect might be partially attributed to mild hyperthermia induced by the laser illumination. 

The study of V. Ziegler et al. entitled “*Photosensitizer adhered to cell culture microplates induces phototoxicity in carcinoma cells*” discusses the phototoxic effect of adherence of photosensitizers on the surfaces of cell culture microplates. Using two rather lipophilic and two hydrophilic substances currently employed in PDT, the authors show that lipophilic photosensitizer molecules adhered to microplates are able to induce cytotoxicity in A431 human epidermoid carcinoma cells upon illumination, independently of whether the photoactive molecule relocalizes from the surface into cells or not. This effect is negligible for hydrophilic photosensitizers. The ability of plastic materials to (reversibly) store photosensitizers might, according to V. Ziegler et al. represent a new approach for photosensitizer delivery or development of antimicrobial coatings.

Inactivation of microorganisms within skin or tissue based on photosensitizers activated by illumination may be complicated by the relatively low penetration depth of visible light. As an alternative to light illumination, F. Nakonechny et al.'s “*Sonodynamic excitation of rose bengal for eradication of Gram-positive and Gram-negative bacteria*” excited the photosensitizer Rose Bengal with 28 kHz ultrasound. The results presented in the study demonstrate for the first time that the novel “sonodynamic” activation of Rose Bengal is able to reduce the number of viable *Staphylococcus aureus* and *Escherichia coli* by up to 4 log units. Sonodynamic treatment represents a promising approach for curing internal infections and sterilization.

In a comprehensive review article entitled “*Innovative strategies to overcome biofilm resistance*” by A. Taraszkiewicz et al. strategies to combat bacterial microfilms are discussed. If organized in biofilms (e.g., in or on humans), bacteria are much more resistant to antimicrobial therapies, including Photodynamic Inactivation. The authors suggest two innovative approaches to increase the bactericidal effect of PDI, namely, the employment of enzymes that affect the biofilm or a combination of PDI with antibiotics, plant extracts, or other biofilm disrupting substances. Application of PDI together with enzymes specific for microbial structures might furthermore increase the selectivity of phototoxicity and/or allow for the usage of lower photosensitizer concentrations. 

Patients suffering from the genetically induced skin disorder epidermolysis bullosa (EB) are exposed to an increased skin tumor risk. P. Larisch et al.'s “*In vitro analysis of photosensitizer accumulation for assessment of applicability of fluorescence diagnosis of squamous cell carcinoma of epidermolysis bullosa patients*” investigated the applicability of fluorescence diagnosis of hypericin and endogenous protoporphyrin IX using an *in vitro* model system, consisting of different (EB) skin cell lines exposed to normal and proinflammatory conditions. A proinflammatory milieu simulated by addition of TNF-alpha appeared not to influence photosensitizer accumulation in skin cells. Carcinoma cells of recessive dystrophic EB showed lower hypericin or PpIX-induced fluorescence than nonmalignant recessive dystrophic EB cells. Further experiments will be necessary to evaluate the possible application of fluorescence diagnosis for early cancer detection of EB patients.

Two-photon absorption is a nonlinear optical process, by which two photons are absorbed simultaneously, thus allowing for doubling the wavelength of excitation of a given photosensitizer in PDT, thereby increasing the penetration depth of the PDT illumination light in tissue and allowing for treatment of thicker malignancies. In this issue, K. Ogawa and Y. Kobuke through their paper “*Two-photon photodynamic therapy by water-soluble self-assembled conjugated porphyrins*” review a set of studies on two-photon PDT with water-soluble and self-assembling conjugated porphyrins. Based on data of two-photon cross section values obtained by open-aperture Z-scan measurements, singlet oxygen production and phototoxicity towards cancer cells *in vitro*, the conjugated porphyrins are suggested as candidates for two-photon PDT agents.

In the paper entitled “*Killing effect of Ad5/F35-APE1 siRNA recombinant adenovirus in combination with hematoporphrphyrin derivative-mediated photodynamic therapy on human nonsmall cell lung cancer*,” L. Xia and coworkers report on the PDT efficiency and the molecular mechanism of cytotoxicity of the Ad5/F35-APE1 siRNA recombinant adenovirus in combination with hematoporphyrin derivative (HpD) in A549 human lung adenocarcinoma cells. Infection of cancer cells with the Ad5/F35-APE1 siRNA recombinant adenovirus significantly reduced the HpD-PDT-induced expression of the APE1 protein and could therefore enhance the photokilling of HpD-mediated PDT.

Three papers discuss the interconnection between PDT and the host's immune response. The methodology report entitled “*Effective combination of photodynamic therapy and imiquimod 5% cream in the treatment of actinic keratoses: three cases*” of L. Held et al. evaluate the therapeutic effect of combining PDT using 5-aminolevulinic-acid-(ALA-) induced PPIX as photosensitizer with imiquimod 5% cream for the clinical treatment of actinic keratoses. Imiquimod is known to regulate the level of cytokines and thereby to influence the immune response and was applied to the treatment area two weeks after PDT. Although a rather small population of patients was part of the pilot study, the results are promising and should be studied in subsequent larger clinical trials: PDT followed by imiquimod treatment was well tolerated and combination of both improved the reduction of actinic keratoses, as demonstrated by the authors. 

Photodynamic treatment of actinic keratoses and nonmelanoma skin cancer in solid organ transplant recipients as well as alternatives to PDT are reviewed by C. Wlodek et al.'s “*Use of photodynamic therapy for treatment of actinic keratoses in organ transplant recipients*”. It is well known that long-term immunosuppressive medication performed on organ transplant recipients increases the risk to develop skin cancers and/or their precursors. The authors present PDT as efficient, safe, and well-tolerated treatment option for these malignancies in immunosuppressed patients but point out that early and numerous treatments on a regular basis are necessary to obtain an optimal response and to prevent development of new actinic keratoses.

E. Panzarini et al. in their paper “*Immunogenic cell death: can it be exploited in photodynamic therapy for cancer?*” review the undoubted key role of the immune response triggered by PDT for the control of cancer cells revival after an antitumor treatment. By elucidating the role of damage associated molecular patterns (DAMPs) at the crossroad between cancer cell death and immunogenicity in PDT, the convincing advantage of PDT over several other cancer treatment options, which is to potentially elicit a specific antitumor immunity, is discussed.

The process of scientific development of new applications of PDT is still highly active and viable. The finalizing contribution of this special issue is therefore an invitation to other researchers to join the successful photodynamic community. T. Kiesslich and coworkers in the paper entitled “*A comprehensive tutorial on in vitro characterization of new photosensitizers for photodynamic antitumor therapy and photodynamic inactivation of microorganisms*” present a comprehensive tutorial on the initial experimental procedures *in vitro*, which help to identify possible applications of newly synthesized photosensitizers in the frame of PDT/PDI. The article covers a detailed description of the experimental approach to the characterization of the photochemical and photophysical properties of a photosensitizer, its uptake kinetics into cells, intracellular localization, and photodynamic action in both, tumor cells and microorganisms. The authors hope that the special issue on PDT in general and the tutorial article in particular will help to stimulate the efforts to expand the convincing benefits of photodynamic procedures within both established and new fields of applications.

